# The LAV‐BPIFB4‐Platelet‐CD47 Axis: A Novel Mechanism Associated With Immune Resilience in Longevity

**DOI:** 10.1111/acel.70602

**Published:** 2026-06-25

**Authors:** Elena Ciaglia, Roberta Maria Esposito, Valentina Lopardo, Francesco Montella, Cristina Basile, Roberta Longo, Anna Maciag, Giuseppe Rescigno, Antonio Damato, Francesco Del Plato, Alfonso Finizio, Carmine Vecchione, Albino Carrizzo, Annibale Alessandro Puca

**Affiliations:** ^1^ Department of Medicine and Surgery “Scuola Medica Salernitana” University of Salerno Fisciano Italy; ^2^ Cardiovascular Research Unit IRCCS Multimedica Milan Italy; ^3^ Clinical Pathology Unit AOU San Giovanni di Dio e Ruggi d'Aragona Salerno Italy; ^4^ Vascular Physiopathology Unit IRCCS Neuromed Pozzilli Italy; ^5^ ASL Salerno‐Ospedale di Comunità di Roccadaspide Salerno Italy; ^6^ Transfusion Medicine Unit‐ ASL Salerno‐Battipaglia Hospital Battipaglia Italy

**Keywords:** CD47, immunomodulation, LAV‐BPIFB4, longevity, monocyte, platelets

## Abstract

Long‐living individuals (LLIs) possess remarkable genetic resilience, characterized by protective variants that confer immune robustness and resistance to age‐related diseases. The longevity‐associated variant of BPIFB4 (LAV‐BPIFB4), enriched in centenarians, demonstrated pleiotropic benefits including reduced inflammation, cardiovascular protection, and immune system rejuvenation. However, the molecular mechanisms underlying these protective effects remain incompletely understood. Here, we revealed that LAV‐BPIFB4 fundamentally reshaped the immune features of platelets to establish enhanced immunomodulatory capacity through CD47 upregulation. Of note, centenarians displayed an elevated percentage of circulating CD47^+^ reticulated platelets (RPs), a condition mimicked by LAV‐BPIFB4 carriers which exhibited significantly elevated CD47 levels both on RPs and mature platelets' surface. In agreement with an early acquirement of CD47 overexpression, MEG‐01 megakaryoblastic cells overexpressing LAV‐BPIFB4 produced CD47‐high platelet‐sized particles. Functionally, platelets from LAV carriers suppressed monocyte activation and inflammatory cytokine production through CD47‐dependent mechanisms, selectively reducing p38 MAPK activation while leaving NF‐κB signaling largely unaffected in response to LPS. rhLAV‐BPIFB4 administration in vivo increased CD47 on murine platelets and reduced, ex vivo, LPS‐induced monocyte activation, validating cross‐species therapeutic potential. Critically, rhLAV‐BPIFB4 protein phenocopies genetic effects, rapidly increasing CD47 expression on wild‐type platelets through cytoskeleton‐dependent trafficking mechanisms and conferring enhanced anti‐inflammatory capacity. This might represent a translatable strategy to replicate some of the biological features associated with a longevity‐associated variant beyond genetic carriers. Our findings establish the platelet‐CD47‐monocyte axis as a pivotal pathway for healthy aging and reveal recombinant LAV‐BPIFB4 as a promising therapeutic approach for managing inflammaging and cardiovascular disease by harnessing the immune‐tuning capacity characteristic of exceptional longevity.

## Graphical abstract

Centenarians display elevated CD47^+^ reticulated platelets, a trait recapitulated in LAV‐BPIFB4 carriers on total platelet pool. LAV‐BPIFB4 drives an early acquirement of CD47 surface expression, enabling platelets to suppress monocyte IL‐6 release. rhLAV‐BPIFB4 phenocopies the effects in non‐genetic carriers, establishing the platelet–CD47–monocyte axis as a therapeutic target against inflammaging.

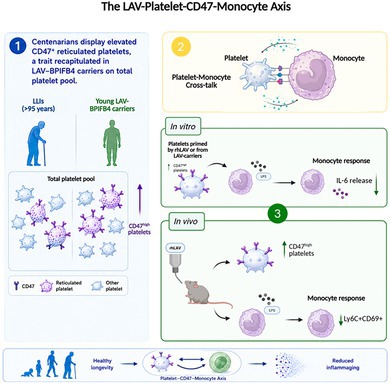

Long‐living individuals (LLIs) possess exceptional genetic resilience with common and rare protective variants and robust immune function. Our laboratory identified that LLIs are enriched for the homozygous longevity‐associated variant (LAV) haplotype in BPIFB4 gene, showing reduced susceptibility to chronic diseases and healthier aging (Villa, Carrizzo, et al. 2015; Villa, Malovini, et al. 2015). LAV‐BPIFB4 reduces inflammation (Ciaglia et al. 2020, 2019; Puca et al. 2020), modulates immunity (Ciaglia et al. 2024, 2020, 2025), provides cardiovascular protection (Cattaneo et al. 2023; Puca et al. 2020), attenuates neuroinflammation (Cattaneo et al. 2022; Di Pardo et al. 2020), and rejuvenates immune system and vasculature (Ciaglia et al. 2022). However, critical gaps remain in understanding how this genetic advantage translates into immune resilience. Multiple pathways are involved, including efficient PKC alpha/PERK phosphorylation of BPIFB4 triggering CXCR4‐mediated eNOS activation (Puca et al. 2020; Villa, Carrizzo, et al. 2015). LAV homozygous carriers show BPIFB4‐enriched platelets, essential for LAV therapeutic effects (Ciaglia et al. 2024). Recent evidence shows that platelets regulate immune modulation and vascular homeostasis (Semple et al. 2011), with centenarian platelets exhibiting distinctive properties (Rabini et al. 2003). Notably, circulating platelets interact with monocytes in a CD47‐dependent manner to limit TLR/NF‐κB signaling and inflammation (Li et al. 2024).

The central question addressed here is whether platelets from centenarians are enriched in CD47, if this is recapitulated in LAV‐BPIFB4 carrier platelets, and whether these features can be harnessed to modulate dysregulated immune systems. To this end, using flow cytometry, we profiled circulating platelets of *N* = 10 LLIs (median‐age 96.5; age range: 95–100) and *N* = 60 controls stratified by age (median‐age 38.7; age range: 24–70) and genotype (as detailed in Figure 1B,C).

**FIGURE 1:**
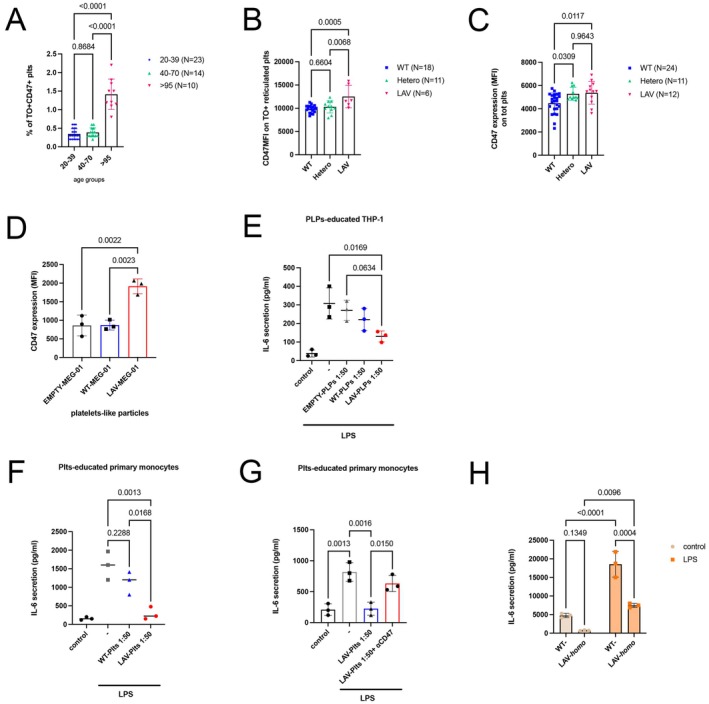
BPIFB4 and CD47 leave an immunoregulatory signature on platelets. Bar graphs reporting the (A) percentage ± SD of CD47^+^ *Thiazole Orange* TO^+^, CD41^+^/CD61^+^ gated platelets stratified by age (group 20–39 *n* = 23; group 40–70 *n* = 14; group > 95 *n* = 10). Eta squared (*η* ^2^): 0.803; (B) the MFI values ± SD of CD47 among TO^+^, CD41^+^/CD61^+^ gated platelets stratified by WT‐ (*n* = 18), Hetero‐ (*n* = 11) or LAV‐BPIFB4 (*n* = 6) genotype_Eta squared (*η* ^2^): 0.30; (C) the MFI values ± SD of CD47 among total CD41^+^/CD61^+^ platelets stratified by WT‐ (*n* = 24) Hetero‐ (*n* = 11) or LAV‐BPIFB4 (*n* = 12) genotype_Eta squared (*η* ^2^): 0.22; due to the small sample size in some groups, normality was assessed using the Shapiro–Wilk test. Groups that met the normality assumption (*p* > 0.05) were analyzed using parametric tests. Statistical significance was determined by one‐way ANOVA with Tukey's HSD post hoc test for multiple comparisons. (D) CD47 surface expression (MFI values ± SD) on platelet‐like particles from parental, EMPTY‐, WT‐BPIFB4‐, and LAV‐BPIFB4‐tranduced MEG‐01 cells from three independent experiments. (E) IL‐6 levels (pg/mL) in THP‐1 supernatants after LPS stimulation following co‐culture with platelet‐like particles from three independent experiments. (F) IL‐6 levels in primary monocyte supernatants after LPS stimulation following co‐culture with WT or LAV‐BPIFB4 PrP from three independent experiments. (G) Effect of CD47 blockade (Magrolimab, 10 μg/mL) on IL‐6 secretion in monocytes co‐cultured with LAV‐BPIFB4 PrP from three independent experiments; (H) IL‐6 secretion (pg/mL) by WT‐ or LAV‐BPIFB4–genotyped monocytes after LPS stimulation. Results were expressed as the mean ± SD of three independent experiments conducted in duplicate using different donors; two‐way ANOVA followed Šídák's multiple comparisons test.

We observed a highly significant percentage of CD47^+^ Thiazole Orange+ (TO^+^) reticulated platelets (RPs)‐ newly released from the bone marrow‐ in LLIs compared to other age categories (1.41% ± 0.40% in > 95 vs. 0.38% ± 0.12% in 40–70 and 0.34% ± 0.14% in 20–39 year‐olds) (Figure 1A) Interestingly, in young carriers (median‐age 38.7; age range: 24–70) this increase is not confined to RPs when stratified by genotype; indeed both RPs and overall totalCD41^+^/CD61^+^ platelets from LAV‐BPIFB4 carriers exhibited significantly increased CD47 expression compared to WT donors (MFI: 12,522 ± 2410 of LAV vs. 9852 ± 801 of WT in the reticulated compartment, and 5373 ± 978 vs. 4474 ± 879 among total platelets, respectively) (Figure 1B,C), establishing CD47 as a key distinguishing feature of LLIs' platelets and as a trait further promoted by LAV‐BPIFB4 genotype. MEG‐01 cells are a megakaryoblastic cell line widely used for the spontaneous releases of platelet‐like particles (PLPs) into the culture medium and for sporadic cytoplasmic processes that make it comparable to megakaryocyte proplatelets (Lopardo et al. 2023; Takeuchi et al. 1991). MEG‐01 lentivirus‐transduced with LAV‐BPIFB4 generated PLPs with significantly higher CD47 levels (MFI: 1915 ± 199) than empty vector (MFI: 861.7 ± 278.7) or WT‐BPIFB4 (MFI: 871 ± 131.6) (Figure 1D), suggesting that longevity variant may influence platelet phenotype at early stages of megakaryocyte differentiation; whether this reflects a genuine ontogenetic mechanism in primary megakaryopoiesis remains to be established. PLPs from LAV‐BPIFB4‐overexpressing cells suppressed THP‐1 monocyte activation in co‐culture (1:50 ratio), showing marked reduction in LPS‐induced IL‐6 release as compared to empty cells (130.3 ± 29.5 vs. 270 ± 54.2 pg/mL) (Figure 1E). Similarly, human primary CD14^+^ monocytes co‐cultured with LAV‐BPIFB4 donor platelets showed significantly decrease in IL‐6 secretion upon LPS stimulation as compared to WT platelets (283.7 ± 172.2 vs. 1137 ± 310.8 pg/mL) (Figure 1F), and the use of Magrolimab (10 μg/mL), an antibody targeting theCD47‐SIRPα interfacereversed this anti‐inflammatory effect (225 ± 106 vs. 632.7 ± 130.8 pg/mL) (Figure 1G), proving that LAV platelet immune‐tuning capacity is CD47‐dependent; whether SIRPα participates in this signaling axis remains to be determined Ex vivo, monocytes isolated from LAV‐homozygous carriers, thus exposed to CD47‐high circulating platelets, displayed attenuated IL‐6 release (7463 ± 555.9 LAV vs. 18,543 ± 3443 pg/mL WT) indicating chronic immune tuning (Figure 1H). Mechanistically, platelets from LAV‐homozygous carriers co‐culture decreased monocyte p38 MAPK activity while preserving NF‐κB signaling (as indicated by sustained p‐P65 levels) in response to LPS, in comparison with WT platelets. This point to a selective action at the post‐transcriptional level (p38‐dependent) without interfering with the transcription of inflammatory genes (NF‐κB dependent). Consistent with this, analysis of AUF‐1, a key regulator of IL‐6 messenger stability, revealed that LAV platelet co‐culture selectively upregulate this mRNA degrading enzyme (Libermann and Baltimore 1990; Paschoud et al. 2006) (Figure 2A). In vivo data utilizing recombinant protein highlighted that platelets from rhLAV‐BPIFB4‐treated mice showed increased CD47 compared to rhWT‐ mice (66,185 ± 23,786 vs. 21,865 ± 24,121) (Figure 2B). Ex vivo validation confirmed that Ly6C^+^ monocytes from rhLAV‐BPIFB4 group displayed attenuated LPS responses in term of % of active Ly6C^+^ CD69^+^ versus rhWT‐ monocytes (2.73% ± 1.75% vs. 6.47% ± 1.67%) (Figure 2C), supporting the chronic exposure to CD47‐high LAV platelets as a cellular tool to blunt aberrant monocytes inflammatory response. Notably, rhLAV‐BPIFB4 protein phenocopies genetic LAV‐BPIFB4 effects. Indeed, platelets from non‐carrier donors exposed to rhLAV‐BPIFB4 (18 ng/mL) exhibited a transient increase in CD47 surface level, peaking at 40 min post‐stimulation (Figure 2D), showing that platelets can acquire the antigenic signature of LAV carriers after rh treatment. Functionally, platelets pretreated with rhLAV‐BPIFB4 conferred enhanced anti‐inflammatory capacity to co‐cultured monocytes, reducing LPS‐induced IL‐6 secretion, an effect abolished by CD47 blockade (Figure 2E). Since CD47 associates with actin and its membrane expression depends on lipid raft organization (Mordue et al. 2017), we hypothesized actin involvement in CD47 membrane exposure by LAV‐BPIFB4. Preincubation with Cytochalasin B significantly reduced rhLAV‐mediated CD47 surface abundance and reversed reduction in LPS‐induced monocytes IL‐6 secretion (Figure 2F,G). Similar results with dynasore further suggest that actin reorganization and membrane dynamics are necessary for LAV‐BPIFB4 to establish the CD47‐high phenotype through a mechanism that remains to be fully elucidated.

**FIGURE 2:**
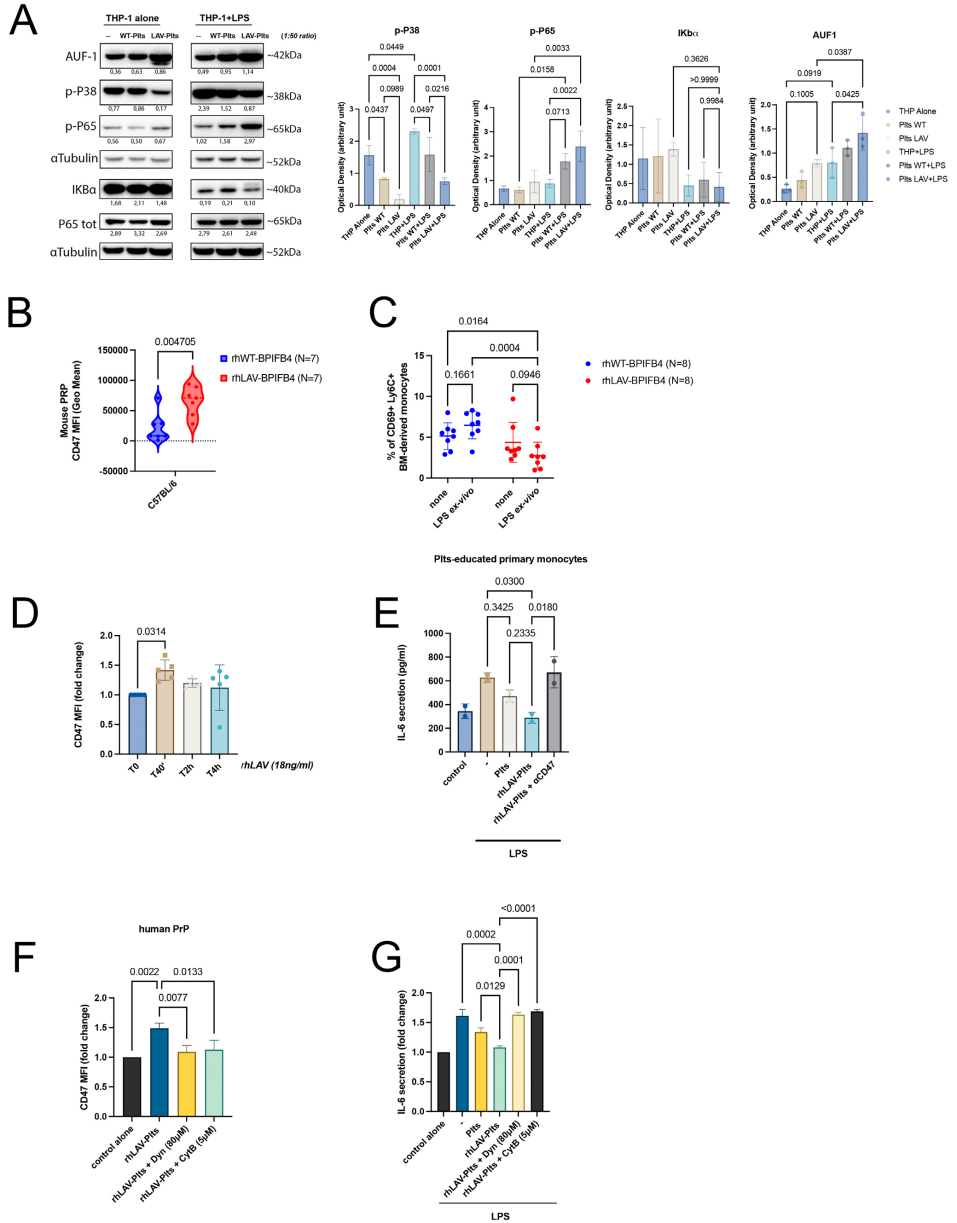
In vitro and in vivo CD47‐mediated effect of recombinant LAV‐BPIFB4. (A) Representative immunoblots for AUF‐1, IκB‐α, p‐p38, p‐p65, p65 in THP‐1 cells following co‐culture with platelets from WT or LAV‐BPIFB4 donorsand LPS stimulation were shown. Bar graphs on the right report aggregated quantification across all three independent biological replicates, presented as mean ± SD (ANOVA). (B) Violin plots showing the MFI values ± SD of CD47 among CD61^+^ gated platelets in the PrP of C57BL/6 treated mice (rhWT‐BPIFB4, *N* = 7; rh‐LAV‐BPIFB4, *N* = 7); before performing multiple unpaired *t*‐test with Holm‐Ṧídák's correction, data normality was confirmed by QQ‐plot visual check for small sample size. (C) Frequency of CD69^+^ Ly6C^+^ monocytes isolated from *N* = 8 rhWT‐BPIFB4‐ and *N* = 8 rhLAV‐BPIFB4‐treated mice after ex vivo LPS stimulation. Statistical analysis by two‐way ANOVA with post hoc Fisher's Least Significant Difference (LSD) test was conducted. Numbers above square brackets show unadjusted LSD *p*‐values. (D) Bar graphs report the time‐course of CD47 surface expression on human platelets (MFI values ± SD) treated with rhLAV‐BPIFB4 (18 ng/mL), expressed as fold change versus basal (T0); from five independent experiments using different donors; one‐way ANOVA with Tukey's HSD post hoc test for multiple comparisons. (E) IL‐6 secretion (pg/mL) by primary monocytes co‐cultured with rhLAV‐BPIFB4–treated platelets in the presence or absence of CD47 blockade (Magrolimab, 10 μg/mL). (F) CD47 surface expression (MFI values ± SD) on rhLAV‐BPIFB4–treated platelets in the presence or absence of Cytochalasin B (5 μM) or Dynasore (80 μM). (G) IL‐6 secretion (pg/mL) by primary monocytes co‐cultured with rhLAV‐BPIFB4–treated platelets in the presence or absence of Cytochalasin B or Dynasore. Results in (E–G) were expressed as the mean ± SD of three independent experiments. Before performing the ANOVA with Tukey's HSD post hoc test, data normality was confirmed by QQ‐plot visual check for small sample size.

For the first time, we show that a subset of CD47^+^ reticulated platelets is uniquely enriched in LLIs, suggesting that this young platelet population exploits a “don't eat me signal” (Mutombo Menga et al. 2025) not only to escape rapid elimination but also to putatively promote longevity. Of note, total‐platelet CD47 does not change appreciably across age (Figure S3), as expected for a protective feature that characterizes the LLIs cohort. Interestingly, in young carriers, this favorable profile is achieved by harboring the LAV‐BPIFB4 variant—a longevity‐associated genetic signature capable of driving increased CD47 expression not only on RPs but overall, in total CD41^+^/CD61^+^ platelets. While the elevated CD47^+^ reticulated‐platelet compartment observed in LLIs is consistent with the effects of LAV‐BPIFB4 demonstrated in genotyped cohorts, direct causal attribution awaits studies in which LLI participants are stratified by BPIFB4 genotype. One possible, though untested, explanation for this apparent discrepancy is that preferential clearance of CD47‐low aged platelets in LLIs may enrich the circulating pool for CD47‐high reticulated forms, leaving total‐platelet CD47 unchanged. Nevertheless, our findings establish platelet CD47 as a critical molecular mediator through which LAV‐BPIFB4 exerts anti‐inflammatory effects, explaining immune resilience and extended health span in carriers. These results align with evidence that platelet‐monocyte CD47‐SIRPα interactions limit TLR signaling and restrain inflammatory responses. Previous observations that platelet depletion abolishes vascular and immunological benefits of systemic AAV‐LAV‐BPIFB4 gene therapy (Ciaglia et al. 2024) underscore platelets' central role, now attributable to CD47‐dependent mechanisms.

Our findings demonstrate platelet CD47 regulates monocyte responses to pathogen‐associated molecular patterns, representing a previously unappreciated mechanism for systemic immune homeostasis. This may explain why platelet counts and function decline with age, potentially contributing to inflammaging through reduced CD47‐mediated immune restraint. Recent discoveries that platelet‐derived factors reverse cognitive decline (Leiter et al. 2023; Park et al. 2023) and rejuvenate aged hematopoietic systems (Zhang et al. 2025) support the paradigm that platelets critically regulate healthy aging beyond hemostatic functions. Our identification of the LAV‐BPIFB4‐CD47 axis adds another dimension, suggesting that interventions targeting platelet immunomodulatory capacity could promote health span.

Chronic low‐grade inflammation (inflammaging) and immune dysregulation fundamentally drive cardiovascular and age‐related diseases. Our findings suggest that platelet CD47 expression could serve as both biomarker and therapeutic target, though validation in larger cohorts is needed. LAV‐BPIFB4's ability to enhance platelet CD47 expression and restrain monocyte‐driven inflammation may in part explain cardiovascular protection, reduced atherosclerosis, improved endothelial function, and delayed cardiac aging observed in previous studies. By limiting excessive monocyte activation, LAV‐BPIFB4‐enhanced platelets maintain balance between protective immune surveillance and pathological inflammation, a characteristic of healthy aging.

The most clinically significant finding is that rhLAV‐BPIFB4 protein treatment can replicate distinctive characteristics of longevity‐associated platelets in individuals lacking the favorable genotype. This protein‐based approach serves as a pharmacological mimetic of the longevity‐associated platelet phenotype, offering a route to translate immune‐tuning capacity observed in LLIs to individuals with inflammaging or cardiovascular disease. While comprehensive safety studies are essential and our sample size was limited, this study provides critical mechanistic insight into how a longevity‐associated genotype translates into improved immune homeostasis. Targeting the platelet‐CD47‐monocyte axis may represent a potential therapeutic strategy for promoting healthy aging and reducing cardiovascular disease. In this context, future rhLAV‐BPIFB4 development could offer a promising avenue toward replicating some of the biological features associated with a variant linked to lifespan in young, healthy persons who are not genetic carriers.

## Author Contributions

E.C. designed and coordinated the research team and wrote the brief report. V.L., F.M., R.M.E., and R.L., conducted ex vivo experiments and FACS analysis. A.D. and A.C. were involved in animal model. C.B. and A.M. established MEG‐01 cell lines and performed ELISA assays. G.R., F.D.P., and A.F. cared for the subjects of the study and evaluation of their health status. C.V. contributed to the critical discussion. A.A.P. supervised and revised the project in its entirety and provided financial support. All authors approved the final version to be published.

## Funding

This work was supported by University of Salerno funding (Codice ORSA254275; PI: Elena Ciaglia); furthermore A.A.P. was supported by the MUR FIS‐2024‐03953 *Advanced Grant* (Fondo Italiano per la Scienza—3rd Edition) and by the Italian Ministry of Health Ricerca Corrente to the IRCCS MultiMedica.

## Ethics Statement

All animal study were performed according to approved protocols by the Istituto Superiore di Sanità, Rome (766/2018‐PR) and were conducted according to EU Directive 2010/63/EU on animals used for scientific purposes. The collection of human samples was approved by the Ethics Committee of Campania 2 for the project “T.V.B. Tromboembolismo venoso e BPIFB4: correlazione tra i livelli di espressione di BPIFB4 e il rischio cardiovascolare in pazienti naïve” (prot. ASLSA‐0281308‐2024 del 23‐12‐2024) and by the IRCCS MultiMedica ethical committee.

## Conflicts of Interest

The authors declare no conflicts of interest.

## Supporting information


**Figure S1:** Example of gating strategy for platelets from human Platelet enriched plasma (PRP) sample.
**Figure S2:** The panel show three independent immunoblots for AUF‐1, IkB‐a, p‐p38, p‐p65, p65 expression in THP‐1 cells, in presence or absence of LPS, following co‐culture with platelets isolated from 3 different WT or 3 different LAV‐BPIFB4 donors.
**Figure S3:** Analysis of CD47 MFI on total circulating platelets from LLIs compared with *n* = 37 adult volunteers grouped in middle‐aged (20–39 years, *n* = 23) and old(er) (40–70 years, *n* = 14) controls with no apparent diseases, who underwent routine preventive laboratory tests.
**Figure S4:** Human PrP from 2 different donors were stimulated with rhLAV‐BPIFB4 (18 ng/mL) for 40 min.
**Data S1:** Supplementary materials and methods.

## Data Availability

The data that support the findings of this study are available on request from the corresponding author. The data are not publicly available due to privacy or ethical restrictions.

## References

[acel70602-bib-0002] Cattaneo, M. , A. P. Beltrami , A. C. Thomas , et al. 2023. “The Longevity‐Associated BPIFB4 Gene Supports Cardiac Function and Vascularization in Ageing Cardiomyopathy.” Cardiovascular Research 119, no. 7: 1583–1595. 10.1093/cvr/cvad008. 36635236 PMC10318395

[acel70602-bib-0003] Cattaneo, M. , A. Maciag , M. S. Milella , E. Ciaglia , A. Bruno , and A. A. Puca . 2022. “Longevity‐Associated Variant of BPIFB4 Confers Neuroprotection in the STHdh Cell Model of Huntington Disease.” International Journal of Molecular Sciences 23, no. 23: 15313. 10.3390/ijms232315313. 36499641 PMC9737551

[acel70602-bib-0004] Ciaglia, E. , V. Lopardo , F. Montella , et al. 2022. “Transfer of the Longevity‐Associated Variant of BPIFB4 Gene Rejuvenates Immune System and Vasculature by a Reduction of CD38^+^ Macrophages and NAD^+^ Decline.” Cell Death & Disease 13, no. 1: 86. 10.1038/s41419-022-04535-z. 35087020 PMC8792139

[acel70602-bib-0005] Ciaglia, E. , V. Lopardo , F. Montella , R. M. Esposito , A. Damato , and A. A. Puca . 2025. “In Vivo Evidence Supports the Effectiveness of the Longevity‐Associated Protein LAV‐BPIFB4 in Reducing Adipose Tissue‐Derived Mediators of Systemic Inflammation to Prevent Vascular Insult and Atheromatous Change.” Adipocyte 14, no. 1: 2580152. 10.1080/21623945.2025.2580152. 41138223 PMC12584601

[acel70602-bib-0006] Ciaglia, E. , F. Montella , A. Carrizzo , et al. 2024. “The Longevity‐Associated BPIFB4 Gene Guarantees Vascular Homeostasis and Immune Protection Through Platelets.” Geroscience 46, no. 6: 6347–6359. 10.1007/s11357-024-01242-9. 38884925 PMC11493904

[acel70602-bib-0007] Ciaglia, E. , F. Montella , V. Lopardo , et al. 2020. “Circulating BPIFB4 Levels Associate With and Influence the Abundance of Reparative Monocytes and Macrophages in Long Living Individuals.” Frontiers in Immunology 11: 1034. 10.3389/fimmu.2020.01034. 32547549 PMC7272600

[acel70602-bib-0008] Ciaglia, E. , F. Montella , A. Maciag , et al. 2019. “Longevity‐Associated Variant of BPIFB4 Mitigates Monocyte‐Mediated Acquired Immune Response.” Journals of Gerontology, Series A, Biological Sciences and Medical Sciences 74, no. S1: S38–S44. 10.1093/gerona/glz036. 31074771

[acel70602-bib-0009] Di Pardo, A. , E. Ciaglia , M. Cattaneo , et al. 2020. “The Longevity‐Associated Variant of BPIFB4 Improves a CXCR4‐Mediated Striatum‐Microglia Crosstalk Preventing Disease Progression in a Mouse Model of Huntington's Disease.” Cell Death & Disease 11, no. 7: 546. 10.1038/s41419-020-02754-w. 32683420 PMC7368858

[acel70602-bib-0010] Leiter, O. , D. Brici , S. J. Fletcher , et al. 2023. “Platelet‐Derived Exerkine CXCL4/Platelet Factor 4 Rejuvenates Hippocampal Neurogenesis and Restores Cognitive Function in Aged Mice.” Nature Communications 14, no. 1: 4375. 10.1038/s41467-023-39873-9. PMC10432533 37587147

[acel70602-bib-0011] Li, C. , S. K. Ture , B. Nieves‐Lopez , et al. 2024. “Thrombocytopenia Independently Leads to Changes in Monocyte Immune Function.” Circulation Research 134, no. 8: 970–986. 10.1161/CIRCRESAHA.123.323662. 38456277 PMC11069346

[acel70602-bib-0012] Libermann, T. A. , and D. Baltimore . 1990. “Activation of Interleukin‐6 Gene Expression Through the NF‐Kappa B Transcription Factor.” Molecular and Cellular Biology 10, no. 5: 2327–2334. 10.1128/mcb.10.5.2327-2334.1990. 2183031 PMC360580

[acel70602-bib-0013] Lopardo, V. , F. Montella , R. M. Esposito , et al. 2023. “SARS‐CoV‐2 Lysate Stimulation Impairs the Release of Platelet‐Like Particles and Megakaryopoiesis in the MEG‐01 Cell Line.” International Journal of Molecular Sciences 24: 4723. 10.3390/ijms24054723. 36902151 PMC10003077

[acel70602-bib-0014] Mordue, K. E. , B. R. Hawley , T. J. Satchwell , and A. M. Toye . 2017. “CD47 Surface Stability Is Sensitive to Actin Disruption Prior to Inclusion Within the Band 3 Macrocomplex.” Scientific Reports 7: 2246. 10.1038/s41598-017-02356-1. 28533511 PMC5440412

[acel70602-bib-0015] Mutombo Menga, A. , X. Hong , and L. Zhu . 2025. “CD47 Signaling in Aging and Age‐Related Diseases: Mechanisms, Challenges, and Therapeutic Opportunities.” Biogerontology 27, no. 1: 27. 10.1007/s10522-025-10370-4. 41428241

[acel70602-bib-0016] Park, C. , O. Hahn , S. Gupta , et al. 2023. “Platelet Factors Are Induced by Longevity Factor Klotho and Enhance Cognition in Young and Aging Mice.” Nature Aging 3, no. 9: 1067–1078. 10.1038/s43587-023-00468-0. 37587231 PMC10501899

[acel70602-bib-0017] Paschoud, S. , A. M. Dogar , C. Kuntz , B. Grisoni‐Neupert , L. Richman , and L. C. Kühn . 2006. “Destabilization of Interleukin‐6 mRNA Requires a Putative RNA Stem‐Loop Structure, an AU‐Rich Element, and the RNA‐Binding Protein AUF1.” Molecular and Cellular Biology 26, no. 22: 8228–8241. 10.1128/MCB.01155-06.4. 16954375 PMC1636780

[acel70602-bib-0018] Puca, A. A. , A. Carrizzo , C. Spinelli , et al. 2020. “Single Systemic Transfer of a Human Gene Associated With Exceptional Longevity Halts the Progression of Atherosclerosis and Inflammation in ApoE Knockout Mice Through a CXCR4‐Mediated Mechanism.” European Heart Journal 41, no. 26: 2487–2497. 10.1093/eurheartj/ehz459. 31289820 PMC7340354

[acel70602-bib-0019] Rabini, R. A. , A. Vignini , D. Martarelli , et al. 2003. “Evidence for Reduction of Pro‐Atherosclerotic Properties in Platelets From Healthy Centenarians.” Experimental Gerontology 38, no. 4: 367–371. 10.1016/s0531-5565(02)00268-1. 12670623

[acel70602-bib-0020] Semple, J. W. , J. E. Italiano Jr. , and J. Freedman . 2011. “Platelets and the Immune Continuum.” Nature Reviews. Immunology 11, no. 4: 264–274. 10.1038/nri2956. 21436837

[acel70602-bib-0021] Takeuchi, K. , M. Ogura , H. Saito , M. Satoh , and M. Takeuchi . 1991. “Production of Platelet‐Like Particles by a Human Megakaryoblastic Leukemia Cell Line (MEG‐01).” Experimental Cell Research 193, no. 1: 223–226. 10.1016/0014-4827(91)90560-h. 1995298

[acel70602-bib-0022] Villa, F. , A. Carrizzo , C. C. Spinelli , et al. 2015. “Genetic Analysis Reveals a Longevity‐Associated Protein Modulating Endothelial Function and Angiogenesis.” Circulation Research 117, no. 4: 333–345. 10.1161/CIRCRESAHA.117.305875. 26034043 PMC5496930

[acel70602-bib-0023] Villa, F. , A. Malovini , A. Carrizzo , et al. 2015. “Serum BPIFB4 Levels Classify Health Status in Long‐Living Individuals.” Immunity & Ageing 12: 27. 10.1186/s12979-015-0054-8. 26675039 PMC4678610

[acel70602-bib-0024] Zhang, S. , C. E. Ayemoba , A. M. Di Staulo , et al. 2025. “Platelet Factor 4 Regulates Hematopoietic Stem Cell Aging.” Blood 146, no. 23: 2765–2778. 10.1182/blood.2024027432. 40920871 PMC12574973

