# Supplementary material for: The LAV‐BPIFB4‐Platelet‐CD47 Axis: A Novel Mechanism Associated With Immune Resilience in Longevity
Source: Aging Cell. 2026 Jun 25;25(7):e70602. doi: 10.1111/acel.70602 (PMC13295143; doi:10.1111/acel.70602)
Supplement: Supplementary file 1 — Figure S1: Example of gating strategy for platelets from human Platelet enriched plasma (PRP) sample. Figure S2: The panel show three independent immunoblots for AUF‐1, IkB‐a, p‐p38, p‐p65, p65 expression in THP‐1 cells, in presence or absence of LPS, following co‐culture with platelets isolated from 3 different WT or 3 different LAV‐BPIFB4 donors. Figure S3: Analysis of CD47 MFI on total circulating platelets from LLIs compared with n = 37 adult volunteers grouped in middle‐aged (20–39 years, n = 23) and old(er) (40–70 years, n = 14) controls with no apparent diseases, who underwent routine preventive laboratory tests. Figure S4: Human PrP from 2 different donors were stimulated with rhLAV‐BPIFB4 (18 ng/mL) for 40 min. Data S1: Supplementary materials and methods. [file ACEL-25-e70602-s001.zip › acel70602-sup-0001-Supinfo1@Final Supplem M&M on line_CLEAN.docx]

**The LAV-BPIFB4-Platelet-CD47 Axis: A Novel Mechanism Underlying Immune Resilience in Longevity.**

Elena Ciaglia,^1,2*^ PhD, Roberta Maria Esposito,^1^ MSc, Valentina Lopardo,^1^ PhD, Francesco Montella,^1^ PhD, Cristina Basile,^1^ MSc, Roberta Longo,^1^ MSc, Anna Maciag,^2^ PhD, Giuseppe Rescigno,^3^ MD, Antonio Damato^4^, Francesco Del Plato^5^, Alfonso Finizio^6^, Carmine Vecchione^1,4^ MD, Albino Carrizzo^1,4^ PhD, Annibale Alessandro Puca,^1,2^ MD.

^1^Department of Medicine and Surgery “Scuola Medica Salernitana”, University of Salerno, Italy

^2^Cardiovascular Research Unit, IRCCS Multimedica, Milan, Italy

^3^Clinical Pathology Unit, AOU San Giovanni di Dio e Ruggi d’Aragona, Salerno, Italy

^4^Vascular Physiopathology Unit, IRCCS Neuromed, Pozzilli, Italy

^5^ASL Salerno-Ospedale di Comunità di Roccadaspide, Salerno, Italy,

6 Transfusion Medicine Unit- ASL Salerno-Battipaglia Hospital, Battipaglia, Italy,

**Supplementary Materials and Methods**

***Human samples***

The study was performed on a group of 70 individuals: n=60 healthy donors (median age 38,7; age range: 24-70; n=35 female, and n=25 male) and 10 long-living individuals LLIs (median-age 96,5; age range: 95-100; n=5 female, and n=5 male) from the exceptional longevity cohort resident in Cilento, a rural area of Southern Italy. N=60 control volunteers’ group was divided in middle-aged (20–39 years) and old(er) group (40–70 years). Each panel reports the analyses on a selected subset of controls (Fig. 1A: n = 37 controls + 10 LLIs; Fig. 1B: n = 35; Fig. 1C: n = 47), reflecting available biological material and genotyping success rates per assay. These were constituted by healthy donors with no apparent diseases who underwent routine preventive laboratory tests at the DEA of Nocera Inferiore, Pagani, and Scafati (Transfusion Center), and stratified by BPIFB4 genotype depending on the experimental purposes. For each donor, two venous blood samples were collected in two different tubes: one with EDTA for monocyte isolation and one with sodium citrate for platelet-rich plasma (PRP) analysis. All participants signed an informed consent for the management of personal anamnestic data and blood samples. Personal and clinical data were processed anonymously, ensuring the protection of privacy and confidentiality of the information collected. The collection of human samples was approved by the Ethics Committee of Campania 2 for the project “T.V.B. Tromboembolismo venoso e BPIFB4: correlazione tra i livelli di espressione di BPIFB4 e il rischio cardiovascolare in pazienti naïve” (prot. ASLSA-0281308-2024 del 23-12-2024) and by the IRCCS MultiMedica ethical committee. The study was conducted in accordance with the ethical principles of the Declaration of Helsinki and current regulations on biomedical research.

***Flow cytometry***

To evaluate CD47 expression levels, platelet-rich plasma (PrP, obtained by centrifuging the vacutainer tube of whole blood with 3.2% sodium citrate at 2200xg for 30 seconds) was stained withan anti-CD47-PE monoclonal antibody (CC2C6, BioLegend; 1 μl x 100 μl of PrP), anti-CD41/61-APC monoclonal antibody (A2A9/6, Sony Biotechnology; 1 μl x 100 μl of PrP), and Thiazole Orange dye (#390062, Sigma-Aldrich, final concentration 1μg/mL in 100 μl of PrP) both at basal conditions and following treatment with recombinant LAV-BPIFB4. After 15 minutes incubation in the dark at room temperature, 400 μl of Staining Buffer (Aurogene, PBS 2% FBS 0.01% Sodium Azide) or paraformaldehyde 1% (at ratio 1:1) was added and cells were immediately analyzed using the FACSVerse and FACSCelesta flow cytometers (BD Biosciences). Similarly, Platelets-like particles (PLPs) released from MEG-01 EMPTY, WT-, and LAV-BPIFB4 expressing cells were labeled with the same anti-CD47-PE monoclonal antibody. Briefly, the different MEG-01 cell lines were harvested and centrifuged at 300xg for 10 minutes. The resulting supernatant (containing the PLPs) was then centrifuged at 800xg for 5 minutes and the pellet obtained was used for CD47 staining. Murine platelet-rich plasma was stained with anti-CD47-FITC monoclonal antibody (miap301, Biolegend, 1 μl x 100 μl of PrP). Bone-marrow derived monocytes from rh-WT- and rhLAV-BPIFB4-treated mice, after ex vivo exposure to LPS (100ng/mL for 24h), were stained with anti-Ly6C-APC-Vio770 (REA796, Miltenyi Biotec) and anti-CD69-PE (REA938, Miltenyi Biotec) for 20 minutes at 4°C in the dark, followed by FACS acquisition and analysis.

***Genotype analysis***

To perform genetic analysis for SNP rs2070235 (p.Ile229Val) on BPIFB4, DNA was extracted from peripheral blood using a specific kit (QIAamp DNA blood midi kit, Qiagen, Düsseldorf, Germany) and following the manufacturer's instructions. The DNA was quantified and the different samples were genotyped using the Taqman rs2070325 probe via RT-PCR.

***Cell lines and culture conditions***

The commercial megakaryoblast cell line MEG-01 (ATCC® CRL-2021) and monocytic THP-1 were cultured in a humidified incubator at 37°C and 5% CO_2_ in RPMI-1640 (Gibco®, Thermo Fisher Scientific) or supplemented with 10% (v/v) fetal bovine serum (FBS, Gibco®, Thermo Fisher Scientific), 1% (v/v) penicillin-streptomycin (Aurogene), 1% (v/v) MEM nonessential amino acids (MEM NEAA, Gibco®, Thermo Fisher Scientific), and 1% (v/v) sodium pyruvate (Aurogene).

***Lentiviral vectors and transfected MEG-01 cell lines***

Lentiviral particles (EMPTY, WT- or LAVBPIFB4) were concentrated by ultracentrifugation (40,000 rpm for 2 hours at 4°C) and stored at -80°C. Lentivirus titration was performed by transducing HEK293T cells with concentrated particles in the presence of 4 μg/ml polybenzene and measuring GFP expression after 3 days by flow cytometry. 500,000 MEG-01 cells were plated in a 12-well plate in RPMI medium and infected with empty lentiviral vectors or particles encoding WT- or LAV-BPIFB4 (at 3 multiplicity of infection (MOI)). After 72 hours, the cells were selected with 1 μg/ml puromycin for 48 hours.

***PLPs-monocyte and Platelet-monocyte co-cultures***

PLPs-monocytes and platelet-monocyte co-cultures were set up by plating monocytes (CD14^+^ isolated from PBMCs or THP-1, a commercial human cell line) in RPMI-1640 culture medium (Gibco®, Thermo Fisher Scientific) supplemented with 10% (v/v) fetal bovine serum (FBS, Gibco®, Thermo Fisher Scientific), 1% (v/v) penicillin-streptomycin (Aurogene), 1% (v/v) MEM nonessential amino acids (MEM NEAA, Gibco®, Thermo Fisher Scientific), and 1% (v/v) sodium pyruvate (Aurogene). Subsequently, PLPs or platelets (PrP) were added at a monocyte:platelet ratio of 1:50, and the co-cultures were incubated overnight at 37°C in a humidified atmosphere with 5% CO₂.

In order to mimic an inflammatory condition *in vitro*, after platelets wash-out (at 800 rpm for 7 min), monocytes were stimulated with lipopolysaccharide (LPS, 100 ng/mL) for 24 h at 37°C in a humidified atmosphere with 5% CO₂. After stimulation, the plate was centrifuged at 2000 rpm for 5 min and the supernatants were stored for subsequent cytokine ELISA assay.

***IL-6 ELISA assay***

To quantify IL-6 released by monocytes following LPS stimulation in the culture medium, culture supernatants were analyzed using the Human IL-6 ELISA kit (Antibodies.com, code A78324), following the manufacturer’s instructions.

***Monocyte isolation***

Peripheral blood mononuclear cells (PBMCs) were isolated from whole blood using density gradient centrifugation with Lymphocyte Separation Medium (Sial, density 1.077 g/ml). Subsequently, the cells were collected and purified in MACS buffer (PBS, 0.5% BSA, 2 mM EDTA) and subjected to positive selection of CD14+ monocytes through immunomagnetic separation (Miltenyi Biotec, Human CD14 MicroBeads UltraPure) following the protocol provided by the manufacturer.

Murine monocytes were isolated from bone marrow through immunomagnetic separation (Miltenyi Biotech, Monocyte Isolation Kit (BM), mouse) following manufacturer’s instructions.

***Drug treatments***

Donor PrPs were treated with recombinant rhLAV-BPIFB4 (18 ng/mL) at different time points (40 min, 2 h, and 4 h) at 37 °C. Where indicated, an anti-CD47 blocking antibody (Magrolimab, Selleckchem.com; 10 µg/mL) was co-administered with rhLAV-BPIFB4. For mechanistic experiments, cells were pre-treated for 30 min at 37 °C with either the actin polymerization inhibitor Cytochalasin B (USBiological; 5 µM) or the dynamin-mediated endocytosis inhibitor Dynasore (Selleckchem.com; 80 µM) prior to rhLAV-BPIFB4 treatment.

***Western Blotting***

THP-1 monocytes conditioned by WT- and LAV-platelets and exposed to LPS 1µg/mL for 1h at 37°C in a humidified atmosphere with 5% CO₂ were washed with PBS (Gibco®, Thermo Fisher Scientific, Waltham, MA, USA), harvested and lysed in ice-cold RIPA lysis buffer containing protease and phosphatase inhibitors. Lysates were cleared by centrifugation (13,000 rpm, 20 min, 4 °C) and protein concentration determined. Equal amounts of protein (~25 μg) were separated by 10% SDS-PAGE and transferred to nitrocellulose membranes. Membranes were blocked with 5% nonfat milk in TBST for 1 h at room temperature and incubated overnight at 4 °C with the following primary antibody: Ab anti-AUF-1 (Abcam, ab259895, Cambridge, United Kingdom), Ab anti-p-p65 (Cell signaling, cs3033, Danvers, MA, USA), Ab anti-p65 (Cell signaling, cs8242, Danvers, MA, USA), Ab anti-IKB-α (Cell signaling, CS9242; Danvers, MA, USA), Ab anti-p-p38 (Cell signaling, CS4511; Danvers, MA, USA) and Ab anti-α-tubulin (Sigma-Aldrich, T6199; St. Louis, Missouri, USA)-conjugated donkey anti-mouse IgG (Bio-Rad) and enhanced chemiluminescence (ECL, Thermo Fisher) and using Azure400 (Azure Biosystems) chemiluminescent imaging system. Bands densitometries were generated using Bio-Rad Image Lab Software.

***In vivo mouse model and sample collection***

All animal study were performed according to approved protocols by the Istituto Superiore di Sanità, Rome (766/2018-PR) and were conducted according to EU Directive 2010/63/EU on animals used for scientific purposes. In this pilot study, we evaluated the effect of rh-WT- and rhLAV-BPIFB4 in an in vivo model. The oral gavage route employed for rhLAV or rhWT-BPIFB4 administration is consistent with prior experimental practice (Alvino et al., 2024; Ciaglia et al., 2025). C57BL/6 mice from Jackson Laboratories were treated with 3µg/mouse of rhWT- or rhLAV-BPIFB4 via gavage each 3 days for 3 weeks. At the end of treatment, mice were anesthetized with ketamine/xylazine, and euthanized by beheading to collect blood and femur in order to harvest bone marrow. Power analysis has been conducted to determine an adequate the number of mice (n=8 rhWT-BPIFB4-treated mice; n=8 rhLAV-BPIFB4-treated mice).

***Statistical analysis***

In all experiments shown, statistical analysis was performed using the GraphPad Prism 10.0 software package for Windows (GraphPad software). For each type of assay or phenotypic analysis, data obtained from multiple experiments were calculated as mean ± standard deviation and analyzed for statistical significance using the t-test and ANOVA test. Before performing the ANOVA and unpaired t test, data normality was confirmed using the Shapiro-Wilk test (p>0.05) for groups with n ≥ 8, and QQ-plot inspection for smaller groups where formal tests lack power.
