# Supplementary material for: The LAV‐BPIFB4‐Platelet‐CD47 Axis: A Novel Mechanism Associated With Immune Resilience in Longevity
Source: Aging Cell. 2026 Jun 25;25(7):e70602. doi: 10.1111/acel.70602 (PMC13295143; doi:10.1111/acel.70602)
Supplement: Supplementary file 1 — Figure S1: Example of gating strategy for platelets from human Platelet enriched plasma (PRP) sample. Figure S2: The panel show three independent immunoblots for AUF‐1, IkB‐a, p‐p38, p‐p65, p65 expression in THP‐1 cells, in presence or absence of LPS, following co‐culture with platelets isolated from 3 different WT or 3 different LAV‐BPIFB4 donors. Figure S3: Analysis of CD47 MFI on total circulating platelets from LLIs compared with n = 37 adult volunteers grouped in middle‐aged (20–39 years, n = 23) and old(er) (40–70 years, n = 14) controls with no apparent diseases, who underwent routine preventive laboratory tests. Figure S4: Human PrP from 2 different donors were stimulated with rhLAV‐BPIFB4 (18 ng/mL) for 40 min. Data S1: Supplementary materials and methods. [file ACEL-25-e70602-s001.zip › Final Supplem Figure Legends on line_CLEAN.docx]

**The LAV-BPIFB4-Platelet-CD47 Axis: A Novel Mechanism Underlying Immune Resilience in Longevity.**

Elena Ciaglia,^1,2*^ PhD, Roberta Maria Esposito,^1^ MSc, Valentina Lopardo,^1^ PhD, Francesco Montella,^1^ PhD, Cristina Basile,^1^ MSc, Roberta Longo,^1^ MSc, Anna Maciag,^2^ PhD, Giuseppe Rescigno,^3^ MD, Antonio Damato^4^, Francesco Del Plato^5^, Alfonso Finizio^6^, Carmine Vecchione^1,4^ MD, Albino Carrizzo^1,4^ PhD, Annibale Alessandro Puca,^1,2^ MD.

^1^Department of Medicine and Surgery “Scuola Medica Salernitana”, University of Salerno, Italy

^2^Cardiovascular Research Unit, IRCCS Multimedica, Milan, Italy

^3^Clinical Pathology Unit, AOU San Giovanni di Dio e Ruggi d’Aragona, Salerno, Italy

^4^Vascular Physiopathology Unit, IRCCS Neuromed, Pozzilli, Italy

^5^ASL Salerno-Ospedale di Comunità di Roccadaspide, Salerno, Italy,

6 Transfusion Medicine Unit- ASL Salerno-Battipaglia Hospital, Battipaglia, Italy,

**Supplementary Figure Legends**

**Supplementary Figure 1**

Example of gating strategy for platelets from human Platelet enriched plasma (PRP) sample.

Platelets are identified as CD41/CD61 positive cells. Positively identified platelets for each tube are then analyzed in another dot plot CD47 versus Thiazole Orange (T.O.) for reticulated platelets or simply CD47 versus CD41/CD61 for mature platelets (right panels).MO/isotype controls for CD47, CD41/CD61 and TO are reported (left panels).

**Supplementary Figure 2**

The panel show three independent immunoblots for AUF-1, IkB-a, p-p38, p-p65, p65 expression in THP-1 cells, in presence or absence of LPS, following co-culture with platelets isolated from 3 different WT or 3 different LAV-BPIFB4 donors.

**Supplementary Figure 3**

Analysis of CD47 MFI on total circulating platelets from LLIs compared with n = 37 adult

volunteers grouped in middle-aged (20-39 years, n = 23) and old(er) (40-70 years, n = 14) controls

with no apparent diseases, who underwent routine preventive laboratory tests.

As reported here, we found no significant differences in CD47 MFI on mature circulating platelets between LLI and the other age groups, suggesting that CD47 levels do not change appreciably across age.

**Supplementary Figure 4**

Human PrP from 2 different donors were stimulated with rhLAV-BPIFB4 (18ng/ml) for 40 minutes. The figure reports the representative immunoblots showing that rhLAV-BPIFB4 treatment increases the BPIFB4 content in human platelets. Bar graphs on the right report aggregated quantification across the two independent biological replicates, presented as mean ± s.d.
