# Supplementary figures and images for: The LAV‐BPIFB4‐Platelet‐CD47 Axis: A Novel Mechanism Associated With Immune Resilience in Longevity
Source: Aging Cell. 2026 Jun 25;25(7):e70602. doi: 10.1111/acel.70602 (PMC13295143; doi:10.1111/acel.70602)

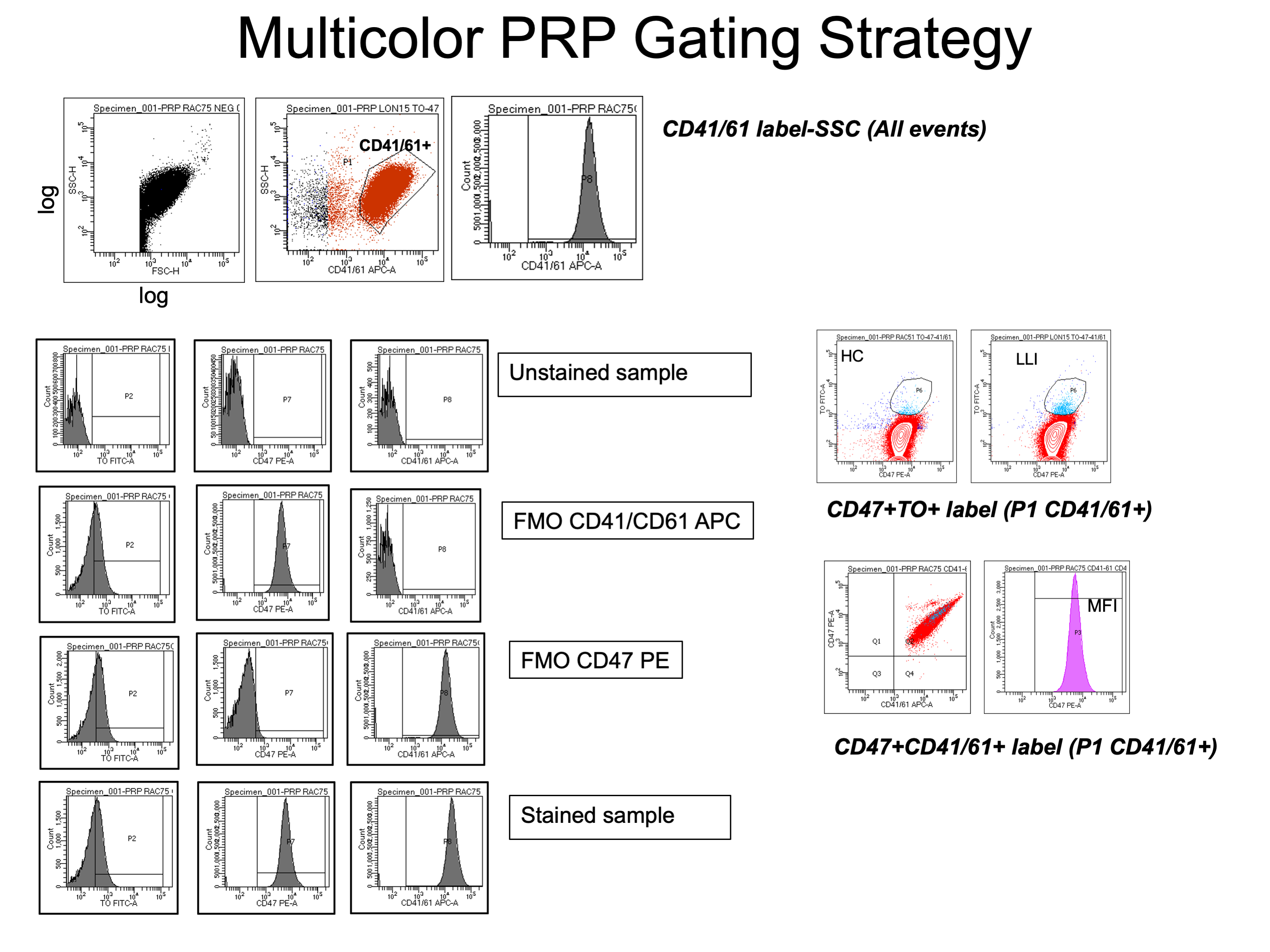

Supplement: Supplementary file 1 — Figure S1: Example of gating strategy for platelets from human Platelet enriched plasma (PRP) sample. Figure S2: The panel show three independent immunoblots for AUF‐1, IkB‐a, p‐p38, p‐p65, p65 expression in THP‐1 cells, in presence or absence of LPS, following co‐culture with platelets isolated from 3 different WT or 3 different LAV‐BPIFB4 donors. Figure S3: Analysis of CD47 MFI on total circulating platelets from LLIs compared with n = 37 adult volunteers grouped in middle‐aged (20–39 years, n = 23) and old(er) (40–70 years, n = 14) controls with no apparent diseases, who underwent routine preventive laboratory tests. Figure S4: Human PrP from 2 different donors were stimulated with rhLAV‐BPIFB4 (18 ng/mL) for 40 min. Data S1: Supplementary materials and methods. [file ACEL-25-e70602-s001.zip › acel70602-sup-0003-FigureS1@Supplementary Figure 1 Gating strategy Aging Cell.tiff]

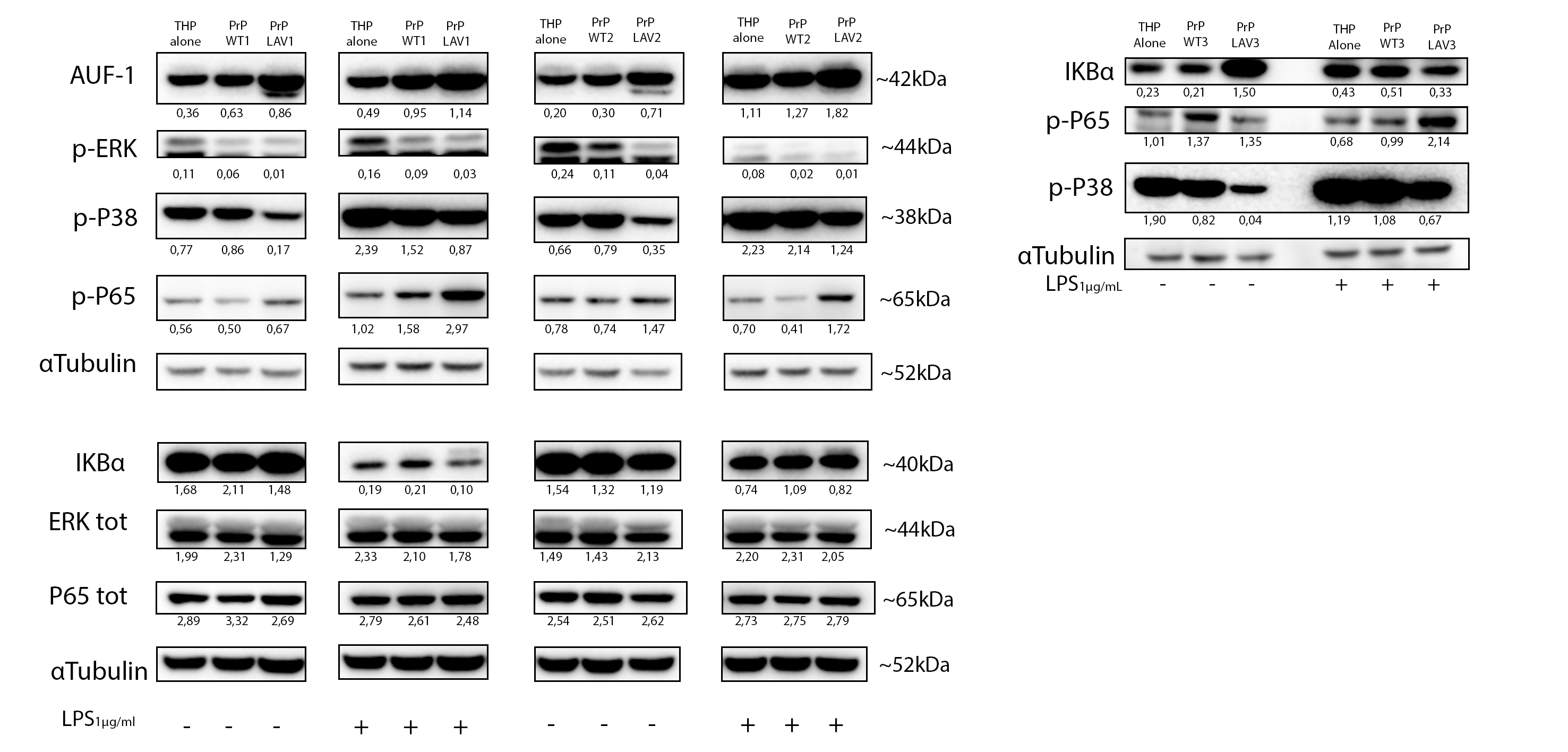

Supplement: Supplementary file 1 — Figure S1: Example of gating strategy for platelets from human Platelet enriched plasma (PRP) sample. Figure S2: The panel show three independent immunoblots for AUF‐1, IkB‐a, p‐p38, p‐p65, p65 expression in THP‐1 cells, in presence or absence of LPS, following co‐culture with platelets isolated from 3 different WT or 3 different LAV‐BPIFB4 donors. Figure S3: Analysis of CD47 MFI on total circulating platelets from LLIs compared with n = 37 adult volunteers grouped in middle‐aged (20–39 years, n = 23) and old(er) (40–70 years, n = 14) controls with no apparent diseases, who underwent routine preventive laboratory tests. Figure S4: Human PrP from 2 different donors were stimulated with rhLAV‐BPIFB4 (18 ng/mL) for 40 min. Data S1: Supplementary materials and methods. [file ACEL-25-e70602-s001.zip › acel70602-sup-0004-FigureS2@Supplementary Figure 2.png]

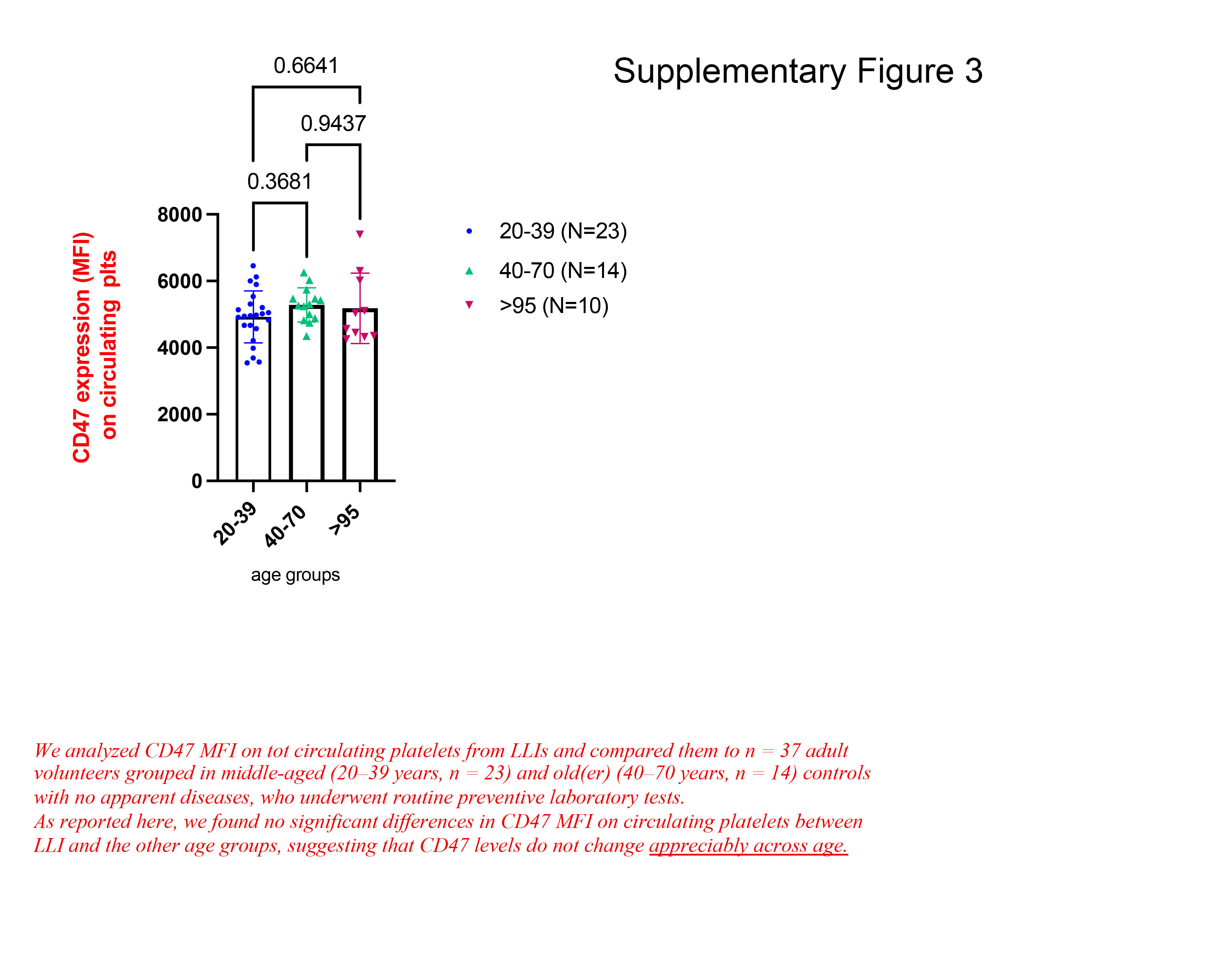

Supplement: Supplementary file 1 — Figure S1: Example of gating strategy for platelets from human Platelet enriched plasma (PRP) sample. Figure S2: The panel show three independent immunoblots for AUF‐1, IkB‐a, p‐p38, p‐p65, p65 expression in THP‐1 cells, in presence or absence of LPS, following co‐culture with platelets isolated from 3 different WT or 3 different LAV‐BPIFB4 donors. Figure S3: Analysis of CD47 MFI on total circulating platelets from LLIs compared with n = 37 adult volunteers grouped in middle‐aged (20–39 years, n = 23) and old(er) (40–70 years, n = 14) controls with no apparent diseases, who underwent routine preventive laboratory tests. Figure S4: Human PrP from 2 different donors were stimulated with rhLAV‐BPIFB4 (18 ng/mL) for 40 min. Data S1: Supplementary materials and methods. [file ACEL-25-e70602-s001.zip › acel70602-sup-0005-FigureS3@Supplementary Figure 3 LQ.jpg]

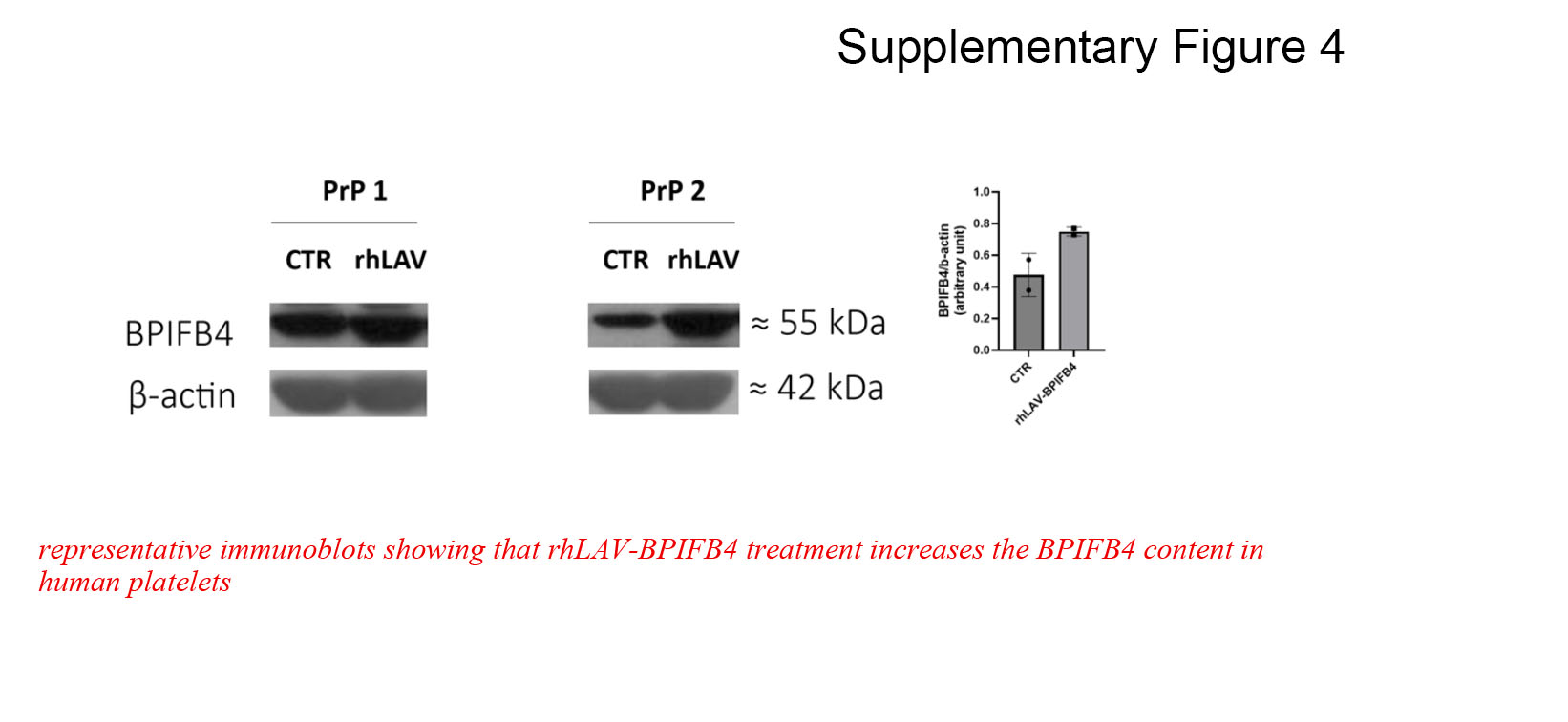

Supplement: Supplementary file 1 — Figure S1: Example of gating strategy for platelets from human Platelet enriched plasma (PRP) sample. Figure S2: The panel show three independent immunoblots for AUF‐1, IkB‐a, p‐p38, p‐p65, p65 expression in THP‐1 cells, in presence or absence of LPS, following co‐culture with platelets isolated from 3 different WT or 3 different LAV‐BPIFB4 donors. Figure S3: Analysis of CD47 MFI on total circulating platelets from LLIs compared with n = 37 adult volunteers grouped in middle‐aged (20–39 years, n = 23) and old(er) (40–70 years, n = 14) controls with no apparent diseases, who underwent routine preventive laboratory tests. Figure S4: Human PrP from 2 different donors were stimulated with rhLAV‐BPIFB4 (18 ng/mL) for 40 min. Data S1: Supplementary materials and methods. [file ACEL-25-e70602-s001.zip › acel70602-sup-0006-FigureS4@Supplementary Figure 4 LQ.jpg]
